# Mermaid Syndrome: Navigating the Challenges of a Rare Congenital Disorder

**DOI:** 10.7759/cureus.71317

**Published:** 2024-10-12

**Authors:** Safina Tanveer, Ayesha Abbas, Maria Leonor Obando Cabezas, Hira Tahir, Tasyoh Thampi

**Affiliations:** 1 Surgery, Khyber Teaching Hospital, Peshawar, PAK; 2 Medicine, Akhtar Saeed Medical and Dental College, Lahore, PAK; 3 General Surgery, Université de Sherbrooke, Sherbrooke, CAN; 4 General Medicine, Dow University of Health Sciences, Karachi, PAK; 5 Internal Medicine, West China School of Medicine, Sichuan University, Chengdu, CHN

**Keywords:** congenital anomaly, mermaid syndrome, neonatal surgery, oligohydramnios, sirenomelia

## Abstract

Sirenomelia, or mermaid syndrome, is a rare congenital disorder characterized by the fusion of lower limbs and often associated with multisystem organ dysfunction, resulting in poor survival beyond the neonatal period. We report a case of sirenomelia in a full-term infant born to a 28-year-old primigravida with no significant medical history, gestational diabetes, or teratogenic exposure. The antenatal period was complicated by oligohydramnios, though routine ultrasounds failed to detect the condition. The diagnosis of sirenomelia was only made after delivery by cesarean section, with a compatible-with-life appearance, pulse, grimace, activity, and respiration (APGAR) score. The infant was referred to the pediatric surgical department due to abdominal distension and fused lower limbs, with plans to manage these conditions if the infant survived long-term. During the three-day hospital stay, vomiting was noted, and a babygram confirmed Stocker and Heifetz type IV sirenomelia and distended large bowel. An exploratory laparotomy revealed gross gastrointestinal and genitourinary abnormalities. A sigmoid colostomy was performed to relieve obstruction. Unfortunately, the infant expired shortly after surgery. This case highlights the challenges of prenatal diagnosis and the limited understanding of surgical management in sirenomelia, particularly given the rarity of survival beyond the neonatal period.

## Introduction

Sirenomelia is a rare congenital anomaly characterized by the fusion of the lower limbs, giving the appearance of a mermaid's tail, which is why it is commonly referred to as mermaid syndrome [1]. Sirenomelia occurs with an incidence of 0.8 to one case per 100,000 births [2]. It is marked by the complete or partial fusion of the lower limbs, renal agenesis, oligohydramnios, an absent urinary tract, missing external genitalia, and an imperforate anus [3]. Tragically, this complex syndrome is often fatal in the neonatal period due to the presence of multiple life-threatening anomalies [4].

The etiology of this condition remains uncertain. Due to its strong resemblance to caudal dysgenesis (CD) and vertebral defects, anal atresia, cardiac abnormalities, tracheoesophageal fistula with esophageal atresia, and renal anomalies (VACTERL) association, it was initially considered a form of caudal regression syndrome [1]. However, the defining anatomical feature that distinguishes sirenomelia from caudal regression syndrome is the presence of a single umbilical artery originating from the vitelline artery. Known risk factors for the syndrome include maternal age under 20 or over 40, maternal diabetes, and prenatal exposure to substances such as retinoic acid, cocaine, and water contaminated by landfills [5].

Ultrasonography is the preferred method for prenatal screening and diagnosis of sirenomelia [3]. Due to its rarity and the limited number of cases that survive the neonatal period, knowledge about the surgical management of sirenomelia remains scarce [6].

This case highlights the relatively extended survival of 56 hours in a full-term infant with type 4 sirenomelia, admitted to the pediatric surgical ward for large bowel obstruction. Given that few cases survive the birth process and early neonatal period, understanding of the surgical management of sirenomelia remains limited. The initial plan was to relieve the obstruction, followed by the surgical separation of the fused legs. Unfortunately, the newborn passed away shortly after surgery, once again leaving the surgical management of sirenomelia an open question.

## Case presentation

We present a rare case of sirenomelia (mermaid syndrome) in a full-term infant, referred to the Pediatric Surgery Department from the Obstetrics and Gynecology (OBGYN) unit of our tertiary care hospital. The infant, a male, was born to a 28-year-old primigravida with an uneventful antenatal history apart from oligohydramnios. The pregnancy, resulting from a non-consanguineous marriage, progressed to 39 weeks and four days and culminated in an elective cesarean section due to breech presentation. The mother, with no known genetic or congenital anomalies in her family, had multiple antenatal visits, where the oligohydramnios was managed with intravenous hydration and betamethasone from 28 to 38 weeks of gestation. Despite regular monitoring, no abnormalities were identified on antenatal ultrasounds, and the parents were not informed of any fetal concerns.

**Figure 1:**
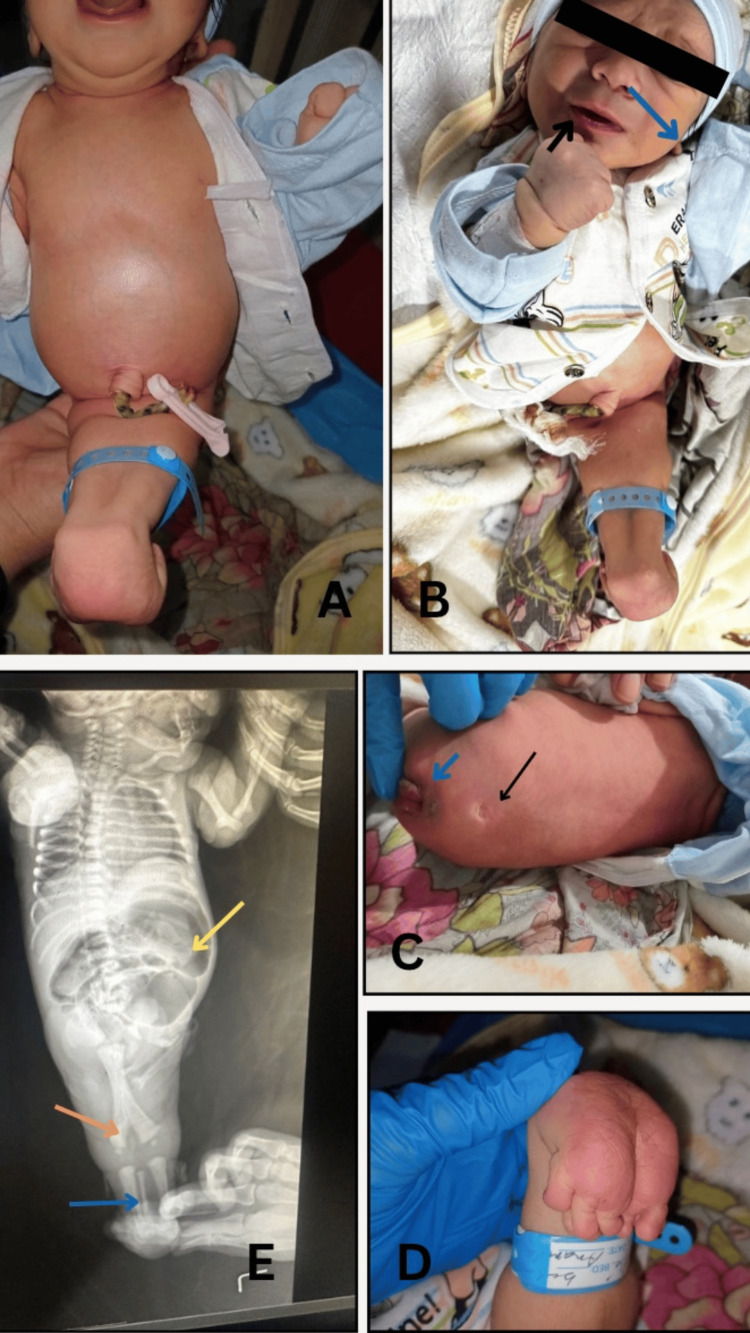
Infant with normal chest, distended abdomen, and fusion of lower limbs from the perineum to the ankle, with feet facing posteriorly (A). Facial features consistent with Potter facies, including a slit-like mouth (black arrow) and low-set ears (blue arrow) (B). Posterior view displaying a flat perineum, a lumbosacral dermal pit (black arrow), and a nonspecified opening with mucosa protruding (blue arrow) (C). Feet fused medially (D). Babygram showing partially fused femurs (orange arrow), two tibias, and an abnormally positioned medial fibula (blue arrow); yellow arrow shows distended large bowel (E)

The infant, with a birth weight of 2.5 kg, had an appearance, pulse, grimace, activity, and respiration (APGAR) score of 6 at one minute and 7 at five minutes, not requiring immediate neonatal intensive care unit (NICU) admission. However, physical examination revealed significant lower body anomalies. The baby had a normal chest, but the abdomen was grossly distended. Features of Potter’s facies were evident, including low-set ears, a broad nasal bridge, prominent lower eyelids, a slit-like mouth, and a receding chin. The upper limbs were normal, but the lower limbs were fused, with feet projecting posteriorly. Pelvic bones and calcaneus were palpable, and both feet had metatarsals, although there were 11 toes in total, six on the right foot, with an accessory toe budding from the fourth toe and five normal toes on the left foot. The umbilical cord contained only one artery and one vein (Figure 1).

A babygram showed a normal ribcage and heart contours, but the spine could not be properly visualized due to excessive bowel gas. The lower femurs were bifid at their distal ends, with two distinct tibial bones and a single medially placed fibula, classifying the case as Stocker and Heifetz type IV sirenomelia. External genitalia were absent, and there was a single opening at the sacral region, later confirmed to be the urethral opening. No anus was present, but a small indentation was noted 2 cm above the urethral opening.

An abdominal ultrasound revealed a normal-sized liver, gallbladder, and spleen. Bilateral kidneys were difficult to visualize due to excessive gut gas, but no other anomalies were initially detected. In the pediatric surgery ward, the infant was otherwise stable except vomiting, for which a nasogastric (NG) tube was passed. An abdominal X-ray confirmed significant large bowel dilation. The patient despite of being on intravenous (IV) fluids had not passed urine by the second day of life. However, urine began to flow from the sacral opening later that day.

The infant was prepared for exploratory laparotomy on the second day of admission. Preoperative blood work revealed an elevated total leukocyte count of 29,900/uL, hemoglobin of 18.4 g/dL, and a platelet count of 254,000/uL. Serum electrolytes indicated a potassium level of 6 mmol/L, sodium at 140.3 mmol/L, and chloride at 111 mmol/L. Urea and creatinine were slightly elevated, at 1.2 mg/dL and 25 mg/dL, respectively. A left lower transverse incision was made, revealing a distended proximal sigmoid colon with a blind, atretic distal end. Hard bilateral masses, suspected to be pelvic kidneys, were noted, but no bladder was palpable. Two pea-sized gonads, resembling testes, were observed posterior to the pubis. A sigmoid colostomy was performed to relieve the obstruction. Unfortunately, the infant experienced a slow recovery from anesthesia and collapsed 12 hours postoperatively. Despite resuscitation efforts, the infant was declared dead on the third day of admission, having survived for 56 hours after birth. The family declined autopsy and karyotyping. This case is reported due to the rarity of sirenomelia and the challenges associated with its surgical management.

## Discussion

Sirenomelia, commonly known as mermaid syndrome, is a rare congenital disorder characterized by the fusion of the lower limbs. This condition is typically associated with multiple organ system dysfunction, which significantly limits survival beyond the neonatal period. Due to its rarity and the low number of survivors, surgical management of sirenomelia remains poorly understood.

In this case, a full-term infant with fused lower limbs was born to a 28-year-old primigravida who had significant oligohydramnios during pregnancy, though the cause was not identified during the antenatal period. Routine ultrasounds in each trimester appeared normal, giving the parents no indication of any congenital abnormalities. The birth was otherwise uneventful, with the newborn achieving an acceptable APGAR score. The baby was referred to the Pediatric Surgery Department after being diagnosed with a large bowel obstruction, confirmed through physical and radiological examination.

The infant was stable and hemodynamically sound and showed no abnormalities in the chest or heart, making them a candidate for surgery to relieve the obstruction. The plan was to address the obstruction first, with the possibility of surgically separating the fused legs or any urogenital anomalies if the child survived long-term. Tragically, the baby passed away shortly after birth, leaving the definitive management of sirenomelia an ongoing challenge in the medical field.

**Figure 2:**
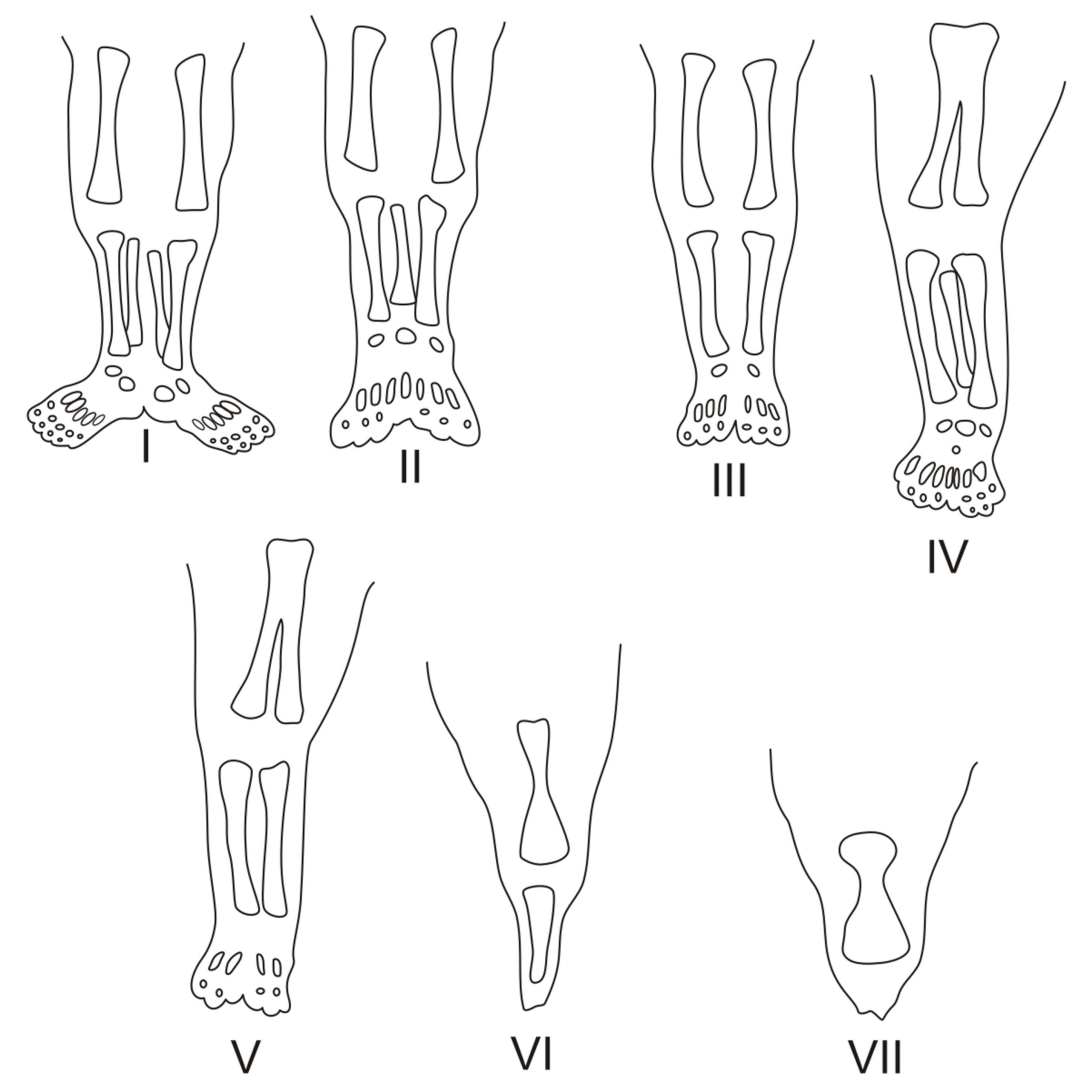
Classification of sirenomelia by the presence or absence of bones within the lower limb. (I) All bones of the thigh and lower leg present. (II) Fused fibula. (III) Fibula absent. (IV) Partially fused femur, fused fibula. (V) Partially fused femur. (VI) Fused femur, fused tibia. (VII) Fused femur, tibia absent This file is freely licensed under the Creative Commons Attribution-Share Alike 4.0 International license. https://en.m.wikipedia.org/wiki/File:Sirenomelia.svg

Sirenomelia can be classified into several categories based on the wide variety of limb malformation phenotypes. The widely used classification is the Stocker and Heifetz method, which has seven types (I-VII) and is based on the presence or absence of the femur, tibia, and fibula (Figure 2) [1]. In our case, the infant was a Stocker and Heifetz type IV with partially fused femurs and a fused fibula.

The exact cause of sirenomelia remains unclear, but two primary theories exist. One is defective blastogenesis, where insufficient mesodermal development leads to impaired formation of caudal structures, suggesting that sirenomelia may represent an extreme form of caudal dysgenesis. The second theory involves abnormal vascular development. In many cases, fetuses with sirenomelia have a single umbilical artery (SUA) originating from the vitelline artery, unlike normal fetuses with two umbilical arteries. Below the SUA’s origin, the aorta becomes narrowed and lacks branches that typically supply the kidneys, intestines, and genitalia. This abnormal blood flow redirects circulation to the placenta, leading to developmental defects in the lower limbs [1]. In our case, examination of the umbilical stump revealed a single umbilical artery and vein, supporting the second theory of sirenomelia involving abnormal vascular development.

Sirenomelia is likely multifactorial, involving various potential causes. Poorly controlled maternal diabetes is associated with sirenomelia, as elevated free oxygen radicals in such cases may contribute to teratogenic effects during embryonic development. Maternal age is a significant risk factor, with both very young (under 20) and older (over 40) ages being associated with increased risk. Certain teratogens, such as retinoic acid, cadmium, and cyclophosphamide, have been linked to sirenomelia in animal studies. Human cases have also been associated with substances like cocaine, landfill water, and certain appetite suppressants. While no chromosomal abnormalities have been linked to sirenomelia in humans, animal models have identified genes such as srn, Tsg1, and Bmp7 that are associated with hindlimb fusion. Global literature includes reports of two cases of sirenomelia within a single family, suggesting a potential genetic basis for the condition. This indicates a Mendelian inheritance pattern, with a 50% chance of incidence in the second generation [7]. Additionally, a postmortem examination of an 18-week male fetus conceived via intracytoplasmic sperm injection (ICSI) revealed the characteristic findings of sirenomelia call for more study to label it as a risk factor [8]. None of the previously mentioned risk factors were present in this couple. The occurrence of mermaid syndrome in this infant appears to have been completely sporadic.

Sirenomelia can be reliably diagnosed in the first trimester via ultrasound, as severe oligohydramnios later in pregnancy, due to urinary tract abnormalities, often hinders visibility [9,10]. In this case, the parents confirmed regular antenatal visits, with routine ultrasounds performed in each trimester and consistent follow-ups with a gynecologist. No congenital anomaly was detected, apart from oligohydramnios, which was managed during the pregnancy. This suggests either the ultrasound report was inaccurate or that it was conducted after significant oligohydramnios had developed, which may have obscured the diagnosis.

## Conclusions

In conclusion, this case underscores the diagnostic challenges of sirenomelia, where routine antenatal ultrasounds may fail to identify congenital abnormalities, particularly in the presence of severe oligohydramnios. Despite regular prenatal care, the condition remained undetected until birth. Additionally, this case highlights the relatively extended survival of 56 hours in a full-term infant with type 4 sirenomelia, admitted for large bowel obstruction. While the initial plan involved relieving the obstruction and considering later leg separation, the infant's passing shortly after surgery reiterates the complexity and uncertainty surrounding the surgical management of sirenomelia. Continued research and case documentation are crucial to improve early diagnosis and potential treatment strategies for this rare condition.
